# Antidiarrheal Effect of 80% Methanol Extract and Fractions of the Roasted Seed of *Coffea arabica* Linn (Rubiaceae) in Swiss Albino Mice

**DOI:** 10.1155/2022/9914936

**Published:** 2022-01-19

**Authors:** Muluken Adela Alemu, Zewdu Birhanu Wubneh, Meaza Adugna Ayanaw

**Affiliations:** ^1^Department of Pharmacy, College of Medicine and Health Sciences, Debre Tabor University, Debre Tabor, Ethiopia; ^2^Department of Pharmacology, School of Pharmacy, College of Medicine and Health Sciences, Gondar University, Gondar, Ethiopia

## Abstract

**Background:**

Globally in 2019, diarrhea was the second leading cause of mortality in children, accounting for more than half a million under-five deaths yearly. Several societies use *Coffea arabica* Linn for the treatment of diarrhea. However, its use is not scientifically validated.

**Objective:**

The study was conducted to evaluate antidiarrheal activity of 80% methanol extract and solvent fractions of roasted seed of *Coffea arabica* Linn in mice.

**Methods:**

*Coffea arabica* Linn seed was roasted, milled, extracted, and fractionated using hexane, ethyl acetate, and distilled water. Castor oil-induced diarrhea, enteropooling, and motility tests were conducted. Effects on onset, number of feces, weight of feces, fluid content, volume and weight of intestinal content, and motility were evaluated by administering 100 mg/kg, 200 mg/kg, and 400 mg/kg of each extract. Negative controls received 10 ml/kg of the vehicle, and positive controls received either loperamide (3 mg/kg) or atropine (1 mg/kg). Data were analyzed using one-way ANOVA followed by Tukey's post hoc test.

**Results:**

Ethyl acetate fraction at all tested doses significantly prolonged (*p* < 0.05) onset of diarrhea. The number and weight of feces were also reduced significantly by crude extract and ethyl acetate fraction. Reduction in fluid content was observed at 200 mg/kg and 400 mg/kg of the crude extract (*p* < 0.01) and aqueous fraction (*p* < 0.001) as well as all tested doses of ethyl acetate fraction (*p* < 0.001). Similarly, the crude extract, ethyl acetate fraction, and aqueous fraction showed a significant reduction in the volume and weight of intestinal content. At 400 mg/kg, the crude extract, hexane fraction, aqueous fraction, and all doses of ethyl acetate fraction showed significant antimotility activity.

**Conclusion:**

The results of this study revealed that the roasted seed of *Coffea arabica* Linn has antidiarrheal activity.

## 1. Introduction

The word “diarrhea” comes from two Greek words “dia” for through and “rheo” for “flow” to mean defecation of liquid stool more than three times a day. In adults, it can be defined as stool weight or volume more than 200 g or 200 ml per day, with increased frequency and passage of loose stools [1]. Globally, it was ranked eighth in causing mortality, accounting for 1.6 million annual deaths in all ages and 2.5 billion episodes, from which about 90% of deaths occur in sub-Saharan Africa and South Asia [2]. Following respiratory tract infections, it was the second leading cause of morbidity and mortality in under-five children worldwide, responsible for 760,000 deaths per year [3]. Diarrhea is caused by noninfectious and infectious agents like bacteria, viruses, parasites, and chronic diseases, from which viruses take the highest allotment causing acute diarrhea [4].

Diarrhea is treated by both traditional and modern medicines. In Ethiopia, since time immemorial, nearly 80% of the population has used plants as a source of medicine, and diarrhea has been one of the most prominent symptoms of diseases treated by traditional medicines [5].

There are several herbal medicines that are claimed for treating diarrhea. *Coffea arabica, Boscia coriacea, Cissampelos pareira, Plumbago zeylanica*, and *Teucrium polium* are some of the medicinal plants utilized in the traditional treatment of diarrhea in many societies [6].

*Coffea arabica* Linn (Bunna in the Amharic language), a plant from the genus *Coffea* and Rubiaceae family, is an evergreen shrub that grows in subtropical and tropical regions [7, 8]. Many *in vivo* and *in vitro* experimental studies on *C. arabica Linn* extracts showed sympathomimetic [9], anti-inflammatory and antioxidant [10], antibacterial and antiviral [11], smooth muscle relaxant [12], suppression of metalloproteinase expression, and improved skin wound healing activities [11].

*Coffea arabica* Linn is a highly rich source of alkaloids, chlorogenic acids, caffeine, and sucrose. In addition, the presence of volatile compounds, such as aldehydes, furfural, ketones, alcohols, esters, formic acid, and acetic acid, was screened [13, 14].

Ethnobotanically, the roasted seed of the plant is used for the treatment of diarrhea in different parts of Ethiopia, including Ghimbi District (Oromia) [15, 16], Butajira (South-central Ethiopia) [17], Wayu Tuka District (East Welega) [18], Gondar Zuria District (Gondar) [19], Chifra (Afar) [20], Libo Kemkem District (Gondar) [21], and North Shoa [22]. Roasting is important for the formation of new chemicals and for physical, structural, and sensorial changes. These changes, occurring due to thermal decomposition and chemical reactions, result in the formation of more than 1000 bioactive compounds [23].

The roasted seed of *Coffea arabica* Linn has activity against *Escherichia coli, Staphylococcus aureus, Enterococcus faecalis, Salmonella choleraesuis, Listeria monocytogenes, Pseudomonas aeruginosa* [24], *Streptococcus mutans, Porphyromonas gingivalis* [25], *Salmonella enterica, Serratia marcescens,* and *Enterobacter cloacae* [26]. In addition, it has shown antiviral activity against *poliovirus* and *herpes simplex virus type 1* [27].

*Coffea arabica* Linn seed is used for both recreational (as stimulant) and medicinal purposes. It is consumed by almost one-third of the world population [28]. As such, even small health benefits or harms could be important on a large population scale.

Even though utilizing traditional medicinal plants for the treatment of many diseases is considered safe and effective, many current scientific evidences have elaborated absence of efficacy for some herbs and the occurrence of toxicity, mutagenicity, carcinogenicity, and drug-herb interactions through inhibition or induction of microsomal enzymes [29, 30]. Current modern drugs used for the treatment of diarrhea are accompanied by many problems, including adverse effects, drug-drug interactions, and contraindications. They are associated with many side effects like constipation, respiratory depression, lethargy, excitement, and coma [31, 32].

In addition, as explained in different ethnobotanical studies, several communities are using the seed of *Coffea arabica* Linn as an antidiarrheal agent in many parts of Ethiopia [15–22]. But the use, efficacy, and safety of the plant were not scientifically validated. Due to these reasons, searching for cheaper, safe, and effective new antidiarrheal medication better than the present drugs is crucial.

## 2. Materials and Methods

### 2.1. Drugs and Chemicals

Distilled water (University of Gondar, Ethiopia), methanol (Sheba Pharma Plc, Ethiopia), loperamide (Medochemie Ltd., Cyprus), castor oil (Amman Pharmaceutical Industries, Jordan), activated charcoal (S.D. Fine Chem Ltd., India), and atropine sulfate injection (Reyoung Pharmaceutical Co., Ltd., China) were used in the experiment.

### 2.2. Plant Material Collection, Identification, and Preparation

After getting permission from the owner of the farmland, a sufficient amount of the fruits of *Coffea arabica* Linn was collected in February 2020 around Teda Town, near Gondar city, Northwest of Ethiopia. The use of the seed of the plant in the present study complies with the IUCN (International Union for Conservation of Nature) Policy Statement on Research Involving Species, which are endangered and at risk of extinction. The plant was authenticated by Dr. Getnet Chekole (a Botanist and Associate Professor of Botanical Science), Department of Biology, College of Natural and Computational Science, University of Gondar, and the specimen (with a voucher number of MA01) was deposited at National Herbarium, Addis Ababa University, Ethiopia.

The normal fresh coffee berry was dried under shade. The seed was hulled from the parchment and then was dried. The dried coffee seeds were washed with water and dried under shade and then were roasted for 14 min at a temperature of 221°C using a laboratory convective hot air oven [33]. The roasted seeds were milled to a coarse powder by using an electrical mill. The powder was kept in the airtight jar until it was used for extraction.

### 2.3. Experimental Animals

The animals were obtained from colonies in the Animal Unit of the Department of Pharmacology, College of Medicine and Health Sciences, University of Gondar. Adult Swiss albino mice of both sexes aged from 8 to 12 weeks and weight ranging from 20 to 30 g were used for the experiment. They were housed in plastic cages with wood chip bedding and had free access to food and water. They were maintained in a room with light having a natural cycle (12 hours on and 12 hours off). The animals were acclimatized in a working laboratory seven days before the start of the experiment. Care and handling of the mice were performed according to OECD (Organization for Economic Cooperation and Development) guideline 420 [34].

### 2.4. Extraction and Fractionation

#### 2.4.1. Preparation of 80% Methanol Crude Extract

The extraction was conducted according to Chalalai et al. [35]. A total of 1.4 kg of *Coffea arabica* Linn seed powder was mixed with 7 liters of 80% hydromethanol in 1 : 5 ratio. Cold maceration technique was used, and the powder was soaked in the solvent for 72 hours at room temperature with frequent intermittent shaking, followed by filtration using a muslin cloth and then Whatman No. 1 filter paper. The marc was reextracted for the second and third time by adding another equal volume of fresh solvent and soaked for 72 hours in each maceration. The filtered extracts were combined and evaporated to dryness at 40°C and then lyophilized. This crude extract was stored in a refrigerator at 4°C until the time of use.

#### 2.4.2. Fractionation

The methanolic crude extract was successively fractionated using different solvents. Eighty grams of the crude extract was suspended in 400 ml of distilled water and poured into a separatory funnel, and then a similar volume of hexane was added and intermittently shaken to extract hexane soluble ingredients. The mixture showed two layers, the water layer being on the bottom. The water extract was collected in a beaker, and another fresh hexane was added to it. Hexane-based extraction was conducted three times separately. All hexane fractions were collected in one beaker. On the remaining residue (aqueous layer), 400 ml of ethyl acetate was added and shaken. The bottom layer was aqueous residue. Ethyl acetate fraction was collected in a beaker. Another 400 ml of ethyl acetate was again added two times separately to get the ethyl acetate fraction. The final residue was an aqueous fraction. The extracts were then dried in an oven at 40°C. The aqueous residue was dried in a lyophilizer. All extracts were kept in an airtight container in a refrigerator until the time of use.

### 2.5. Phytochemical Screening of Seeds of *Coffea arabica* Linn

The presence of secondary metabolites such as flavonoids, tannins, anthraquinones, glycosides, steroids, phenols, terpenoids, alkaloids, and saponins was assessed for the crude extract and solvent fractions using standard screening tests [36].

### 2.6. Animal Grouping and Dosing

To test the presence or absence of antidiarrheal activity, three models were used. For each extract, every model used thirty mice by randomly allocating them to five groups (6 animals per group). Group I was negative control and treated with a vehicle (negative controls for crude extract and aqueous fraction received 10 ml/kg distilled water, whereas negative controls for hexane and ethyl acetate fraction received 10 ml/kg 2% tween 80). Group II was positive control and treated with loperamide 3 mg/kg for the castor oil-induced diarrhea and enteropooling models and atropine sulfate 1 mg/kg for the gastrointestinal motility test model. Animals from Groups III to V were treated with 100 mg/kg, 200 mg/kg, and 400 mg/kg doses of the extracts of *Coffea arabica* Linn. The doses of the extracts were determined as to 100, 200, and 400 mg/kg based on acute toxicity test. That is, one-tenth of 2000 mg/kg of the dose used in acute oral toxicity test is used to determine the middle dose, and one-half of and 2 times the middle dose was used to determine the lowest and highest doses, respectively [37].

### 2.7. Acute Oral Toxicity Studies

Acute toxicity testing was conducted in healthy and nonpregnant female Swiss albino mice since they are slightly sensitive [38]. Swiss albino mice of 8–12 weeks of age were used. They were kept in the laboratory for 7 days prior to the start of dosing to allow for acclimatization to the conditions. All mice were fasted (food but not water) for 4 hours before and 2 hours after the administration of the extract. According to OECD guideline 420, the first mouse was given 2 g/kg of the methanol crude extract. Since no sign of toxicity was observed within 24 hours, the rest four mice were given 2 g/kg of the crude extract and observed every 30 min for 4 hours and daily for 14 days [34] for the presence or absence of signs of toxicity such as hair erection, loss of appetite, tremors, lacrimation, salivation, diarrhea, convulsions, mortality, and other signs of toxicity [39].

### 2.8. Antidiarrheal Activity Determination

#### 2.8.1. Castor Oil-Induced Diarrhea

The method described by Shoba and Thomas [40] was used for this study. For the test, a total of thirty mice of either sex were randomly divided into five groups (six mice per group) and fasted for 18 hours.

Mice were dosed as described in the grouping and dosing section. After one hour, mice were given 0.5 ml of castor oil orally, and then they were individually placed on the floor covered with nonwetting transparent paper. The transparent paper was changed every hour [41].

During an observation period of 4 hours, the time of onset of diarrhea, the number of wet fecal drops, the total number of fecal outputs (frequency of defecation), and the weight of fresh feces were recorded. Onset was measured in minutes starting from the administration of castor oil to the appearance of first diarrheal stool. The number of total feces and number of wet feces in the negative control group were considered as 100%. Percentage of inhibition of diarrhea and percentage inhibition of defecation were then calculated by the following formula [42]:

(1) $$\begin{array}{c} \%\text{ inhibition of diarrhea}=\frac{\text{mean number of wet stools of }\left(\text{negatve control group}-\text{treated group}\right)}{\text{mean number of wet stools of negatve control group}}\times \;100,\\ \%\text{ inhibition of defecation}=\frac{\text{total number of feces in the }\left(\text{negative control}-\text{treated group}\right)}{\text{total number of feces in the negative control}}\times 100. \end{array}$$

#### 2.8.2. Castor Oil-Induced Enteropooling

The method used by Chitme et al. [43] was used to determine the extracts' effect on castor oil-induced intestinal fluid accumulation. The effect of *Coffea arabica* Linn seed extract on intestinal fluid accumulation was determined by measuring the weight and volume of accumulated fluid in the small intestine. For both the extract and each fraction, a total of thirty mice were grouped into five groups, six mice per group, and then deprived of both food and water for 18 hours prior to the experiment. Mice were dosed as described in the grouping and dosing section. Castor oil (0.5 ml) was given through oral gavage one hour after dosing. One hour later, the mice were first anesthetized by ketamine and sacrificed by cervical dislocation, and the intestine was removed after tying the pyloric and cecum ends. Then, the weight of the intestine with its content was measured. Then, the content in the lumen was expelled into a graduated tube, and then the volume was measured [42]. The intestine was reweighed again, and the weight of the full and empty was recorded. Percentage inhibition was calculated as

(2) $$\begin{array}{c} \%\text{ of inhibition by using MWIC}=\frac{\text{MWICC}-\text{MWICT}}{\text{MWICC}}\times 100, \end{array}$$

where MWIC denotes the mean weight of intestinal content, MWICC denotes the mean weight of intestinal content of the control group, and MWICT denotes the mean weight of intestinal content of the test group.

(3) $$\begin{array}{c} \%\text{ of inhibition by using MVIC}=\frac{\text{MVICC}-\text{MVICT}}{\text{MVICC}}\times 100, \end{array}$$

where MVIC denotes the mean volume of intestinal content, MVICC denotes the mean volume of intestinal content of the control group, and MVICT denotes the mean volume of intestinal content of the test group.

#### 2.8.3. Gastrointestinal Motility Test by Charcoal Meal

Each extract needed a total of 30 mice which were assigned into five groups, six mice per group, and deprived of food for 18 hours. Dosing was performed as described in the grouping and dosing section. After one hour, 0.5 ml castor oil was administered to each mouse. Then, one hour after administration of castor oil, 1 ml of charcoal meal (5% charcoal suspension in 2% tween 80) was administered by oral gavage. One hour later, being anesthetized by ketamine, mice were sacrificed by cervical dislocation, the abdomen was opened, the small intestine was removed, and the length was measured with a measuring ruler. Then, the length traveled by charcoal from the pylorus to the cecum was measured and expressed as a percentage of the overall length of the small intestine [44].

(4) $$\begin{array}{c} \text{peristalsis index}\left(\text{PI}\right)=\frac{\text{distance travelled by the charcoal meal}}{\text{total length of small intestine}}\times 100,\\ \%\text{ of inhibition}=\frac{\text{PI of negative control}-\text{PI of drug or extract treated}}{\text{PI of negative control}}\times 100. \end{array}$$

The *in vivo* antidiarrheal index (ADI *in vivo*) was calculated according to the formula developed by Aye-Than et al. [45]:

(5) $$\begin{array}{c} \text{ADI }in\;vivo=\sqrt[{3}]{D\text{freq}*G\text{meq}*P\text{freq}}, \end{array}$$

where *D* freq is the delay in defecation time or diarrhea onset obtained from castor oil diarrhea test, calculated as

(6) $$\begin{array}{c} D\text{freq}=\frac{\text{mean onset of diarrhea }\left(\text{in treated group}-\text{ in the negative control group}\right)}{\text{mean onset of diarrhea in the negative control group}}\times 100. \end{array}$$

*G* meq is the gut meal travel reduction (as % of control) obtained from charcoal meal test (% inhibition), and *P* freq is the reduction in the number of wet stools (as % of the negative control) from the castor oil-induced diarrhea model (% inhibition of diarrhea).

### 2.9. Ethical Consideration

The animals were handled in accordance with the care and use of laboratory animals guidelines [46]. Ethical approval was obtained from the Ethics Committee of the Department of Pharmacology, School of Pharmacy, University of Gondar.

### 2.10. Statistical Analysis

The results were expressed as a mean ± standard error of the mean (SEM) of responses. The results were statistically analyzed using SPSS version 25. The presence or absence of significant differences between groups was assessed by one-way analysis of variance (ANOVA) followed by post hoc Tukey's multiple comparison test. The results were considered significant if *p* value was less than 0.05.

## 3. Results

### 3.1. Phytochemical Screening

As shown in Table 1, many phytochemical constituents except cardiac glycosides were tested positive in the crude extract. Ethyl acetate fraction was able to localize many phytochemicals, whereas hexane fraction allocates few phytochemical constituents (steroids and anthraquinones).

### 3.2. Acute Oral Toxicity Test

The acute oral toxicity test had shown neither visible toxicity nor death in 14 days observation period upon administration of a single dose of 2000 mg/kg of the crude extract in mice. The absence of visible toxicity and mortality at 2000 mg/kg dose indicates that the crude extract has a wider safety margin and midlethal dose greater than 2000 mg/kg in mice.

### 3.3. Determination of Antidiarrheal Activity

#### 3.3.1. Castor Oil-Induced Diarrhea

In the castor oil-induced diarrheal model, 80% methanol crude extract of *Coffea arabica* Linn significantly prolonged the onset of diarrhea and decreased the total number of feces at 400 mg/kg when compared with the negative control (*p* < 0.05). The extract's percentage reduction of the number of wet stools was 38.7% and 45.3% at 200 mg/kg and 400 mg/kg, respectively (*p* < 0.05). Relative to the negative control, the crude extract also significantly lowered the total weight of feces by 46.8% and 48.1% (*p* < 0.01) with the dose of 200 mg/kg and 400 mg/kg, respectively. An apparent difference was observed in the fluid content of feces at 200 mg/kg and 400 mg/kg of the crude extract (*p* < 0.05) when compared with the negative control (Table 2).

Ethyl acetate fraction prolonged diarrhea-free period/onset of diarrhea (*R*^2^ = 0.22) at all tested doses (*p* < 0.01). A significant reduction in the number of total feces was observed at 200 mg/kg and 400 mg/kg of the fraction (*p* < 0.05). The average percentage reduction of the number of wet stools was 50% at 400 mg/kg dose (*p* < 0.01). The weight of total feces was reduced by 36%, 69.3%, and 76% at doses of 100 mg/kg, 200 mg/kg, and 400 mg/kg doses of ethyl acetate fraction, respectively (*R*^2^ = 0.34). An evident difference was observed in the fluid content of feces at all tested doses (*R*^2^ = 0.37) of ethyl acetate fraction compared to the negative control (*p* < 0.001). The fraction had shown comparable effects with loperamide-treated mice on many parameters (onset of diarrhea, total number of feces, and number of wet feces) (Table 2).

When compared with the negative control, the aqueous fraction at a dose of 400 mg/kg prolonged the onset of diarrhea significantly (*p* < 0.01). Moreover, the weight of total feces was inhibited by 45.6% and 60.3% at doses of 200 mg/kg (*p* < 0.01) and 400 mg/kg (*p* < 0.001) of the fraction, respectively. The fraction also showed a significant difference in the fluid content of feces at 200 mg/kg and 400 mg/kg dose (*p* < 0.001) (Table 2).

#### 3.3.2. Effects on Castor Oil-Induced Enteropooling in Mice

The volume of intestinal content was reduced in a dose-dependent manner by 50.7% (*p* < 0.05) and 59.4% (*p* < 0.01) at 200 mg/kg and 400 mg/kg of methanol crude extract when compared with the negative control. In addition, the crude extract reduced the weight of intestinal content significantly by 45.2% (*p* < 0.05) and 56.2% (*p* < 0.01) with a dose of 200 mg/kg and 400 mg/kg, respectively (Table 3).

Compared to the negative control, ethyl acetate fraction reduced the volume of intestinal content by 42.2%, 46.9%, and 48.4% at the doses of 100 mg/kg, 200 mg/kg, and 400 mg/kg (*p* < 0.05). Ethyl acetate fraction was also responsible for 43.5% and 47.8% of weight reduction at 200 mg/kg and 400 mg/kg doses, respectively (*p* < 0.05). For both parameters, no significant difference was observed between ethyl acetate fraction treated and loperamide-treated mice (Table 3).

The aqueous fraction showed lower activity at the highest tested dose. At 400 mg/kg, the volume of intestinal content was reduced by 25.7%, and the weight reduction was 24.3% (*p* < 0.05) (Table 3).

#### 3.3.3. Effects on Castor Oil-Induced Intestinal Transit in Mice

The methanol crude extract and hexane fraction at a dose of 400 mg/kg significantly inhibited gastrointestinal transit of charcoal meal by 20.6% and 20.4%, respectively, when compared with the negative control (*p* < 0.01) (Table 4).

Interestingly, compared to the negative control, ethyl acetate fraction showed significant inhibition at 100 mg/kg (20.1%, *p* < 0.01), 200 mg/kg (37.9%, *p* < 0.001), and 400 mg/kg (45%, *p* < 0.001). The aqueous fraction at 400 mg/kg inhibited gastrointestinal transit of charcoal meal by 15.9% (*p* < 0.05) when compared with the negative control (Table 4).

## 4. Discussion

People customarily use plant preparations intending them to be effective against diarrhea. *Coffea arabica* Linn is traditionally used as an antidiarrheal agent without scientific substantiation [15–22]. Therefore, the objective of this study was to validate the antidiarrheal activity of the roasted seed of *Coffea arabica* Linn in many places.

Acute oral toxicity of 80% methanol crude extract of *Coffea arabica* Linn was determined based on OECD guideline 420 [34]. Administration of doses lower than 2000 mg/kg in mice did not bring serious acute toxicity or death. Due to this, the seed of the plant can be categorized under category 5 based on the Globally Harmonized System of Classification and Labeling of Chemicals (GHSCLC) [38].

In the castor oil-induced diarrhea model, both the crude extract and ethyl acetate fraction had shown significant difference (*p* < 0.05) in the number of wet and total feces, onset of diarrhea, weight of fresh feces, and fluid content when compared with the negative control.

Hexane fraction showed lower or no significant activity in most of the parameters, while ethyl acetate fraction exhibited greater activity in most of the evaluation parameters. The result from this study could suggest that more active phytochemical constituents were medium polar and selectively partitioned in both quantity and/or quality to ethyl acetate (alkaloids, flavonoids, phenols, tannins, saponins, steroids, anthraquinones, and terpenoids), while less active secondary metabolites were solubilized in hexane (steroids, anthraquinones, and terpenoids).

Aqueous fraction showed lower effectiveness against diarrhea even though many active phytochemical constituents are contained in it. One of the plausible reasons for the lower activity of this fraction may be due to the hydrolysis of active agents to inactive chemicals [47]. In addition, less active forms of phytochemicals (glycosides, alkaloids, flavonoids, phenols, tannins, saponins, and glycosides) may be partitioned to it.

Since castor oil induces diarrhea through inhibition of absorption [48] and promotion of secretion and motility [49], the probable mechanism of antidiarrheal activity of the extracts may be due to the presence of different phytochemicals which have a promotive effect on fluid and electrolyte absorption as well as inhibition of secretion and motility. Moreover, reduction of the number of wet feces and significant difference in fluid content in the treated group, especially in ethyl acetate fraction (*p* < 0.001), strengthen extracts' antisecretory activity.

Nonsteroidal anti-inflammatory drugs, apart from inhibition of prostaglandin synthesis, prolong the onset of diarrhea in the castor oil-induced diarrhea model [50]. Likewise, the late onset of diarrhea by *Coffea arabica* Linn extracts may be due to its proven anti-inflammatory activities [10]. Prevention of intestinal secretion may be one of the mechanisms of action of extracts. The crude 80% methanol extract showed significant activity in reducing both weight and volume of intestinal content. Possibly, this may be due to the increased effect of the seed on sympathetic activity [9]. Increased sympathetic nerve activity reduces intestinal secretion [51], while it stimulates intestinal absorption through activation of alpha 2 adrenergic receptors [49]. Flavonoids, which are present in the crude extract and partitioned to ethyl acetate and aqueous fractions, inhibit both the production of prostaglandin E2 and expression of COX-2, which are key regulators of inflammatory processes [52]. Furthermore, flavonoids activate alpha 2 receptors of luminal absorptive cells to stimulate absorption [53]. Hence, the presence of flavonoids could increase intestinal absorption of water, and electrolytes accounted for decreased volume and weight of intestinal content.

Also, the activity may be due to the presence of other phytochemical constituents like steroids, alkaloids, tannins, phenols, and the like. Steroids inhibit the release of PGE-2 and PGI-2 from macrophages, which are key mediators of inflammation [54]. Similarly, alkaloids interfere with the functions of neutrophils and monocytes. They inhibit adherence and locomotion of these cells so that castor oil-induced inflammation cannot be exacerbated more [55]. Tannins effect as an astringent and its anti-inflammatory activity may also be accounting for antidiarrheal activity. They either bind and precipitate or shrink proteins. Tannins also modulate cystic fibrosis transmembrane conductance regulator protein (CFTR), a membrane protein that acts as a channel to transport chloride ions from epithelial cells to the lumen in a way that reduces secretion in the small intestine and colon [56]. In addition, they reduce intestinal secretion by inhibiting intracellular Ca^2+^ inward current [57].

Anthraquinones [58] and terpenoids [59] have anti-inflammatory activity. The anti-inflammatory activity of terpenoids may arise from their ability to inhibit the production of prostaglandin E2 [54]. Moreover, flavonoids, alkaloids, and terpenoids attenuate nitric oxide synthesis, which is activated by ricinoleic acid to increase secretion in the gut [60]. Phenols are known to have antioxidant activity by neutralizing free radicals [10]. Free radicals may cause inflammation. Activation of the inflammatory cascade in turn may cause the synthesis of prostaglandins. Prostaglandins are known to stimulate the secretion of fluid and electrolytes in the small intestine and also decrease the absorption of glucose [3]. The combined effects of these phytochemicals resulted in significant antidiarrheal activity. The activity was dose-dependent, implying that the effect increases as the dose increases.

Away from that, the hexane fraction was devoid of significant activity in reducing intestinal fluid accumulation, and the aqueous fraction had a little activity at the highest tested dose. But ethyl acetate fraction had shown better activity (*p* < 0.05). Many active phytochemical constituents like steroids, flavonoids, alkaloids, tannins, saponins, anthraquinones, phenols, and terpenoids were attracted to ethyl acetate (Table 1). Increased activity of ethyl acetate fraction may be due to synergistic activities of these constituents.

The other mechanism of antidiarrheal agents is the reduction of gastrointestinal motility. In this model, ethyl acetate fraction had shown a significant reduction in the propulsion of charcoal meal at all tested doses (*p* < 0.01). All the other extracts (both the crude and other fractions) had comparable significant effects revealed at the highest tested dose (*p* < 0.01). The antimotility activity may be due to different phytochemical compositions of solvent extracts. Caffeine and other alkaloids, which are constituents of *Coffea arabica* Linn, are known smooth muscle relaxants tested on asthmatic patients [12]. This smooth muscle relaxing activity may account for decreased motility. Likewise, intestinal smooth muscle motility may be reduced by increased sympathetic nerve activity resulting from the consumption of *Coffea arabica* Linn [9, 51].

Deceased motility will allow intestinal contents to stay there for a longer time, which by itself increases absorption. Flavonoids and terpenoids are known to inhibit intestinal motility [53, 61]. Studies revealed that tannins decrease peristaltic movements through inhibition of intracellular Ca^2+^ inward current [57].

The extracts of *Coffea arabica* Linn showed antibacterial activity [11]. This shows that the plant has positive activity on multiple causes of diarrhea (both infectious and noninfectious), making it preferable for the development of new potential antidiarrheal medication.

Antidiarrheal index (ADI) is the measure of combined effects of extracts for *D* freq (delay in diarrhea onset), *G* meq (gut meal motility reduction as % inhibition), and *P* freq (reduction in the number of wet stools as % inhibition). Based on ADI value, the crude extract and fractions are ordered as ethyl acetate fraction > crude extract > aqueous extract > hexane fraction (Table 5). The extract having high ADI indicates its strong antidiarrheal activity [56]. In all of the extracts, ADI increases with dose. The highest antidiarrheal index (ADI) for ethyl acetate fraction implies its superiority in antidiarrheal activity relative to both the crude extract and fractions. On the contrary, the hexane fraction, which showed insignificant antidiarrheal activity in most of the parameters, had scored the lowest ADI. Probably, the absence of phytochemical constituents (alkaloids, flavonoids, phenols, tannins, and glycosides), which may be responsible for the antidiarrheal activity, may account for the scantiness of the effect.

## 5. Conclusion

As to the results of this study, it is possible to wind up that the crude extract, aqueous, and ethyl acetate fractions of the roasted seed of *Coffea arabica* Linn showed promising antidiarrheal activity. Hence, the seed can be a potential source for the development of new antidiarrheal medication. Generally, this finding supports the traditional use of roasted seed of *Coffea arabica* Linn as an antidiarrheal agent.

## Figures and Tables

**Table 1:** Results of phytochemical screening for the extracts of *Coffea arabica* Linn.

Chemical constituents	80% methanol extract	Fractions		
		Ethyl acetate	Hexane	Aqueous residue
Alkaloids	+	+	−	+
Flavonoids	+	+	−	+
Phenols	+	+	−	+
Tannins	+	+	−	+
Saponins	+	+	−	+
Steroids	+	+	+	−
Glycosides	+	−	−	+
Anthraquinones	+	+	+	−
Cardiac glycosides	−	−	−	−
Terpenoids	+	+	+	-

Note: + = present; − = absent.

**Table 2:** Effects of the crude extract and fractions of the seeds *Coffea arabica* Linn on castor oil-induced diarrheal model in mice.

Group and dose	Diarrhea-free period/onset time of diarrhea (min)	Total number of feces	Total number of wet feces in 4 hours	Total weight of fresh feces (g)	Fluid content of feces (ml)	% inhibition of weight	% inhibition of defecation	% inhibition of diarrhea
Control	55.00 ± 5.05	6.67 ± 0.67	5.17 ± 1.47	0.79 ± 0.06	0.62 ± 0.03	—	—	—
80ME 100	75.167 ± 5.3	6.00 ± 0.58	4.33 ± 1.03	0.7 ± 0.08^d1,e1^	0.53 ± 0.07^d1,e1^	11.3	10	16.24
80ME 200	83.50 ± 5.42	5.33 ± 0.49	3.17 ± 1.17^a1^	0.42 ± 0.03^a2,b1,^	0.29 ± 0.02^a2^	46.8	20	38.68
80ME 400	93.00 ± 7.99^a1^	3.83 ± 0.3^a1^	2.83 ± 0.75^a1^	0.41 ± 0.08^a2^	0.27 ± 0.08^a3^	48.1	42.6	45.26
Lop 3	144.33 ± 14.18^a3,c3,d3,e2^	3.33 ± 0.71^a2,c1^	1.33 ± 1.03^a3,c2^	0.12 ± 0.03^a3,c3,d1,e1^	0.02 ± 0.002^a3,c3,d1,e1^	84.8	50	74.27
Control	54.67 ± 3.86	6.83 ± 0.70	5.33 ± 0.49	0.78 ± 0.04	0.61 ± 0.032	—	—	—
HF 100	67.33 ± 8.58	6.17 ± 1.25	4.5 ± 0.96	0.71 ± 0.05	0.54 ± 0.039	9	9.7	15.6
HF 200	73.83 ± 8.59	5.83 ± 0.60	4.17 ± 0.60	0.67 ± 0.13	0.5 ± 0.097	14.1	14.6	21.8
HF 400	77.50 ± 9.71	5.50 ± 0.99	3.83 ± 0.60	0.53 ± 0.10	0.38 ± 0.073	32.1	19.5	28.1
Lop 3	145.50 ± 14.67^a3,c3,d3,e3^	3.17 ± 0.60^a1^	1.33 ± 0.49^a2,c2,d1^	0.12 ± 0.02^a3,c3,d2,e1^	0.02 ± 0.003^a3,c3,d3,e2^	84.6	53.6	75
Control	58.00 ± 4.88	6.67 ± 0.42	5.00 ± 0.37	0.75 ± 0.04	0.64 ± 0.04	—	—	—
EF 100	128.67 ± 11.63^a2^	5.17 ± 0.95	3.33 ± 0.71	0.48 ± 0.09^a1,d1,e1^	0.36 ± 0.08^a3,b3,d2,e3^	36.0	22.5	33.4
EF 200	132.67 ± 11.53^a2^	4.00 ± 0.58^a1^	2.83 ± 0.48^a1^	0.23 ± 0.06^a3^	0.13 ± 0.02^a3^	69.3	40	43.4
EF 400	133.33 ± 13.37^a2^	3.67 ± 0.49^a1^	2.50 ± 0.34^a2^	0.18 ± 0.06^a3^	0.06 ± 0.01^a3^	76	45	50
Lop 3	147.83 ± 15.17^a3^	3.33 ± 0.42^a2^	1.50 ± 0.43^a3^	0.15 ± 0.029^a3,c2^	0.03 ± 0.004^a3,c3^	80	50.1	70
Control	57.50 ± 4.83	6.33 ± 0.56	4.83 ± 0.31	0.73 ± 0.05	0.59 ± 0.04	—	—	—
AF 100	80.50 ± 4.01	6.00 ± 0.58	4.33 ± 0.42	0.66 ± 0.08^d2,e3^	0.52 ± 0.067^d2,e3^	9.6	5.2	10.4
AF 200	87.67 ± 3.85	5.33 ± 0.56	3.50 ± 0.43	0.37 ± 0.05^a2^	0.24 ± 0.05^a3^	45.6	15.8	27.5
AF 400	95.67 ± 8.13^a2^	4.17 ± 0.48	3.33 ± 0.42	0.29 ± 0.05^a3,c3^	0.16 ± 0.04^a3^	60.3	34.1	31
Lop 3	150.17 ± 12.08^a3,c3,d3,e3^	3.17 ± 0.60^a2,c1^	1.33 ± 0.33^a3,c3,d2,e2^	0.13 ± 0.02^a3,c3,d1^	0.02 ± 0.004^a3,c3,d1^	76.0	49.9	72.5

All values are expressed as mean ± standard error of the mean (SEM) (*n* = 6); ^a^compared to the control, ^b^compared to the positive standard, ^c^compared to 100 mg/kg, ^d^compared to 200 mg/kg, ^e^compared to 400 mg/kg, ^1^*p* < 0.05, ^2^*p* < 0.01, and ^3^*p* < 0.001; 80ME = 80% hydromethanolic extract, HF = hexane fraction, EF = ethyl acetate fraction, AF = aqueous fraction, and Lop3 = loperamide 3 mg/kg. Controls received 10 ml/kg distilled water (for AF and 80ME) and 2% tween 80 (for EF and HF).

**Table 3:** Effects of 80% methanol crude extract and solvent fractions of seeds of *Coffea arabica* Linn on the gastrointestinal fluid accumulation in mice.

Group and dose	Volume of intestinal content (ml)	% inhibition	Weight of intestinal content (g)	% inhibition
Control	0.69 ± 0.11	—	0.73 ± 0.11	—
80ME 100	0.55 ± 0.05^b2^	20.3	0.56 ± 0.05^b1^	23.3
80ME 200	0.34 ± 0.08^a1^	50.7	0.4 ± 0.08^a1^	45.2
80ME 400	0.28 ± 0.03^a2^	59.4	0.32 ± 0.03^a2^	56.2
Lop 3	0.17 ± 0.02^a3^	75.4	0.26 ± 0.04^a2^	64.4
Control	0.62 ± 0.1	—	0.68 ± 0.1	—
HF 100	0.55 ± 0.03^b3^	11.3	0.60 ± 0.03	11.8
HF 200	0.44 ± 0.04^b2^	29	0.50 ± 0.04	26.5
HF 400	0.41 ± 0.03^b1^	33.9	0.47 ± 0.03	30.9
Lop 3	0.16 ± 0.03^a3^	74.2	0.21 ± 0.03^a3,c3,d2,e1^	69.1
Control	0.64 ± 0.11	—	0.69 ± 0.11	—
EF 100	0.37 ± 0.03^a1^	42.2	0.44 ± 0.04	36.2
EF 200	0.34 ± 0.02^a1^	46.9	0.39 ± 0.02^a1^	43.5
EF 400	0.33 ± 0.08^a1^	48.4	0.36 ± 0.08^a1^	47.8
Lop 3	0.17 ± 0.02^a3^	73.4	0.21 ± 0.02^a3^	69.6
Control	0.70 ± 0.08	—	0.74 ± 0.08	—
AF 100	0.54 ± 0.03	22.9	0.60 ± 0.04	18.9
AF 200	0.53 ± 0.02	24.3	0.57 ± 0.02	23
AF 400	0.52 ± 0.02^a1^	25.7	0.56 ± 0.03^a1^	24.3
Lop 3	0.17 ± 0.01^a3,c3,d3,e3^	75.7	0.25 ± 0.01^a3,c3,d3,e3^	66.2

All values are expressed as mean ± standard error of the mean (SEM) (*n* = 6); ^a^compared to the control, ^b^compared to the standard, ^c^compared to 100 mg/kg, ^d^compared to 200 mg/kg, ^e^compared to 400 mg/kg, ^1^*p* < 0.05, ^2^*p* < 0.01, and ^3^*p* < 0.001; 80ME = 80% hydromethanolic extract, HF = hexane fraction, EF = ethyl acetate fraction, AF = aqueous fraction, and Lop3 = loperamide 3 mg/kg. Controls received 10 ml/kg distilled water (for AF and 80ME) and 2% tween 80 (for EF and HF).

**Table 4:** Effects of 80% methanol crude extract and solvent fractions of seeds of *Coffea arabica* Linn on gastrointestinal transit in mice.

Group and dose	Mean length of small intestine (cm)	Mean distance traveled by the charcoal meal (cm)	Peristaltic index (%)	% inhibition
Control	49.50 ± 1.61	41.00 ± 1.93	82.67 ± 1.67	—
80ME 100	50.17 ± 1.54	38.33 ± 1.17	76.66 ± 2.84^b3^	7.3
80ME 200	48.17 ± 1.08	36.33 ± 1.58	75.41 ± 2.65^b3^	8.8
80ME 400	47.25 ± 1.39	30.83 ± 1.60^a2,c1^	65.64 ± 4.15^a2,b3^	20.6
Atrop 1	52.17 ± 1.49	20.00 ± 2.19^a3,c3,d3,e2^	38.46 ± 4.16^a3^	53.5
Control	49.67 ± 1.52	43.33 ± 0.88	87.52 ± 2.37	—
HF 100	51.00 ± 0.97	40.83 ± 1.78	79.99 ± 2.69	8.6
HF 200	49.50 ± 0.99	37.83 ± 1.78	76.43 ± 3.37	12.7
HF 400	52.33 ± 1.65	36.33 ± 1.94	69.63 ± 3.76^a2^	20.4
Atrop 1	50.67 ± 0.88	19.00 ± 2.13^a3,c3,d3,e3^	37.60 ± 4.33^a3,c3,d3,e3^	57
Control	50.33 ± 1.54	44.83 ± 1.25	89.14 ± 0.93	—
EF 100	48.83 ± 0.79	34.67 ± 1.94^a2,e2^	71.18 ± 4.41^a2,d1,e3^	20.1
EF 200	53.00 ± 0.93	29.33 ± 1.94^a3^	55.37 ± 3.54^a3^	37.9
EF 400	51.17 ± 0.60	25.00 ± 1.53^a3^	49.02 ± 3.41^a3^	45
Atrop 1	53.50 ± 1.34	18.33 ± 1.48^a3,c3,d3^	34.21 ± 2.43^a3,c3,d3,e1^	61.6
Control	49.83 ± 1.70	42.33 ± 1.09	85.32 ± 3.01	—
AF 100	51.58 ± 1.38	40.00 ± 1.15	77.57 ± 1.29	9.1
AF 200	50.17 ± 0.70	38.83 ± 1.66	77.33 ± 2.61	9.4
AF 400	52.17 ± 1.89	37.50 ± 2.08	71.76 ± 2.44^a1^	15.9
Atrop 1	51.17 ± 1.80	20.67 ± 1.63^a3,c3,d3,e3^	40.95 ± 4.21^a3,c3,d3,e3^	52

All values are expressed as mean ± standard error of the mean (SEM) (*n* = 6); ^a^compared to the control, ^b^compared to the standard, ^c^compared to 100 mg/kg, ^d^compared to 200 mg/kg, ^e^compared to 400 mg/kg, ^1^*p* < 0.05, ^2^*p* < 0.01, and ^3^*p* < 0.001; 80ME = 80% hydromethanolic extract, HF = hexane fraction, EF = ethyl acetate fraction, AF = aqueous fraction, and Atrop 1 = atropine 1 mg/kg. Controls received 10 ml/kg distilled water (for AF and 80ME) and 2% tween 80 (for EF and HF).

**Table 5:** *In vivo* antidiarrheal indices (ADIs) of 80% methanol crude extract and solvent fractions of roasted seed of *Coffea arabica* Linn.

Group	*D* freq (from castor oil-induced diarrhea)	*G* meq (from charcoal meal test)	*P* freq (from castor oil-induced diarrhea)	ADI
Control	—	—	—	—
80ME 100	36.67	7.3	16.24	16.32
80ME 200	51.82	8.8	38.68	26.03
80ME 400	69.1	20.6	45.26	40.09
Posit con	162.42	53.5	74.27	86.42
Control	—	—	—	—
HF 100	23.16	8.6	15.6	14.6
HF 200	35.05	12.7	21.8	21.33
HF 400	41.76	20.4	28.1	28.82
Posit con	166.14	57	75	89.22
Control	—	—	—	—
EF 100	121.84	20.1	33.4	43.41
EF 200	128.74	37.9	43.4	59.6
EF 400	129.88	45	50	66.36
Posit con	154.88	61.6	70	87.41
Control	—	—	—	—
AF 100	40	9.1	10.4	15.59
AF 200	52.47	9.4	27.5	23.85
AF 400	66.38	15.9	31	31.98
Posit con	160.87	52	72.5	84.65

80ME = 80% hydromethanolic extract, HF = hexane fraction, EF = ethyl acetate fraction, and AF = aqueous fraction. Posit con is atropine 1 mg/kg for gastrointestinal motility test and loperamide 3 mg/kg in case of castor oil-induced diarrhea and enteropooling models. Controls received 10 ml/kg distilled water (for AF and 80ME) and 2% tween 80 (for EF and HF). *D* freq, the delay in diarrhea onset obtained from castor oil diarrhea test; *G* meq, the gut meal motility reduction as % inhibition; *P* freq, the purging frequency or reduction in the number of wet stools as % inhibition of diarrhea; ADI, antidiarrheal index.

## Data Availability

The datasets used and/or analyzed during the current study are available from the corresponding author upon reasonable request.
